# Practice to pitch: The relationship between force-velocity profiles and match-day performance of semi-professional rugby union players

**DOI:** 10.3389/fspor.2023.1066767

**Published:** 2023-03-30

**Authors:** Ormond Heather, Patrick Lander, Russell Rayner

**Affiliations:** ^1^ School of Health and Sport Science, Eastern Institute of Technology, Hawke's Bay, New Zealand; ^2^ School of Allied Health, Exercise and Sports Sciences, Charles Sturt University, Port Macquarie, NSW, Australia

**Keywords:** rugby performance indicators, force-velocity profiling, training specificity, force orientation, sled push, sled pull, jammer push-press

## Abstract

**Introduction:**

This exploratory study aimed to assess the relationship between athlete neuromuscular performance and rugby performance indicators. Specifically, the study looked at the force-velocity profiles (FVPs) derived from four common resistance exercises and their relationship with rugby performance indicators (RPIs).

**Methods:**

The study recruited twenty-two semi-professional male rugby players (body mass 102.5 ± 12.6 kg, height 1.85 ± 0.74 m, age 24.4 ± 3.4 years) consisting of ten backs and twelve forwards. Prior to the first game of a Covid-impacted nine-match season, participants performed four common resistance exercises (barbell box squat, jammer push-press, sled pull, and sled push) at incremental loads to establish force-velocity profiles. During the season, rugby performance indicators (post-contact metres, tries, turnovers conceded, tackles, try assists, metres ran, defenders beaten, and tackle breaks) were collated from two trusted sources by a performance analyst. Correlational analyses were used to determine the relationship between the results of FVPs and RPIs.

**Results:**

The study found a statistically significant, moderate, positive correlation between tackle-breaks and sled push *V*_0_ (*r* = .35, *p* = .048). Significant, large, positive correlations were also found between tackles and jammer push-press *V*_0_ (*r* = .53, *p* = .049) and tackle-breaks and sled pull *F*_0_ (*r* = .53, *p* = .03). There was a significant, negative relationship between sled pull *V*_0_ and tackle-breaks (*r* = −.49, *p* = .04). However, the largest, significant correlation reported was between metres ran and sled pull *F*_0_ (*r* = .66, *p* = .03).

**Conclusion:**

The study suggests that a relationship may exist between FVPs of particular exercises and RPIs, but further research is required to confirm this. Specifically, the results suggest that horizontal resistance training may be best to enhance RPIs (tackle-breaks, tackles, and metres ran). The study also found that maximal power was not related to any rugby performance indicator, which suggests that a specified prescription of either force or velocity dominant exercises to enhance RPIs may be warranted.

## Introduction

Rugby is a full-contact, intermittent invasion sport (1). As in many invasion sports, the aim is to move the ball into the opposition's territory and score a goal (2–5). Rugby performance indicators (RPIs) are match statistics that are believed to reflect performance (2, 6). Previous research has investigated RPIs associated with successful teams and postulates that improving RPIs such as tackles made, metres run, and defenders beaten may enhance the team's frequency of success (2, 4).

During competitive rugby matches, the tackle events associated with dominating territory require that players collide, each using their physical capacities to overpower opponents and advance along the field (5, 7–10). During these contact moments, players produce forceful muscle actions in proportion to the movement velocity, as represented by the force-velocity relationship (5, 7–10). Thus, prescribing physical conditioning based upon velocity and strength, may improve physical capacities and rugby performance (5, 7).

Both strength and speed are critical for rugby performance (8–12), particularly in the contact moments associated with the territory-based sport, such as tackling or breaking tackles (5, 7, 11). Recent advancements in force-velocity profiling (FVP) have allowed coaches to better assess athletes' neuromuscular performance. While profiling metrics have been explored in activities such as sprinting and jumping (13–16), more research is needed to fully understand the value of FVP in conditioning for sport-specific tasks in rugby. Research has shown that an athlete's force and velocity characteristics are highly individual (14) and position-specific (17–19) in rugby. For example, backs display more velocity-dominant FVPs (20, 21), whereas forwards are more force-dominant (20, 21). However, there is an opportunity to explore further the relationship between conditioning and match performance, such that by profiling the force-velocity capacities of athletes, coaches may be better able to prescribe athlete-specific and position-specific conditioning programmes.

In addition to the specificity of force and velocity characteristics, rugby coaches should consider movement orientation when programming resistance training. Typically, resistance training in rugby is vertically-orientated (22), with athletes exerting force against gravity. However, rugby performance often requires horizontal movements such as tackling (8, 11, 12, 23). Whilst relationships have been identified between some measures of vertical force production and horizontal sprint performance in training, vertically-oriented exercises may not relate to horizontal-orientated RPIs on match-day performance. Therefore, this exploratory study investigates the relationships between FVPs obtained from four common resistance exercises and match-day RPIs. Examining these relationships may provide coaches with the tools to enhance performance in practice and translate it to the pitch.

## Methods

### Experimental design

A correlational design was used to determine the relationship between FVPs and RPIs in semi-professional rugby players. Profiles were created in four commonly prescribed exercises that range in force orientation (vertical, hybrid of horizontal, and two horizontal orientated exercises respectively) barbell box squat, jammer push-press, sled pull, and sled push. RPIs recorded for this study were; post-contact metres, tries, turnovers conceded, tackles, try assists, metres ran, defenders beaten and tackle breaks. Data were recorded over a Covid-impacted nine-match competitive season.

### Participants

The study recruited twenty-two semi-professional provincial-level male rugby players from the Hawke's Bay region of New Zealand (age 24.4 ± 3.4 years, body mass 102.5 ± 12.6 kg, height 1.85 ± 0.074 m). Playing positions were distributed as backs (*n* = 10) and forwards (*n* = 12). Six players were in their first year of open-grade professional rugby. All players had two or more years of experience with resistance training, either through school or rugby organisation (provincial grade or higher). The testing was conducted 12 days before the competition season commenced. All athletes were medically screened before testing. Those deemed medically unfit did not participate in the study.

### Neuromuscular testing

Testing occurred indoors in a temperature-controlled training facility in Napier, New Zealand. The barbell box squat and jammer-push-press were performed on rubber matting using equipment familiar to all athletes. The sled pull and sled push were performed on indoor synthetic grass with minimal wear. Athletes wore running shoes in good condition during testing. Testing commenced following a 15 min standardised warm-up using a RAMP protocol (24).

Known weights and a Linear Position Transducer (LPT) (GymAware, Kinetic Performance Technology, Canberra, Australia) connected to an iPad (Apple, California, Untied States) were used to assess force and velocity in the barbell box squat and jammer-push-press. A load cell (PT100LC, PT Limited, Auckland, New Zealand) and timing lights (Swift Duo, Swift Performance, Queensland, Australia) were used to assess force and velocity in the sled pull and sled push (Xtreme Elite Prowler sled, Elite Fitness, Auckland, New Zealand). The four resistance exercises used were selected as they are all common to rugby conditioning programmes. Athletes rotated around the testing stations in groups of four, completing the tests in a consistent pre-determined order with approximately seven minutes of rest between each individual's trials. All testing was conducted in a single session.

### Assessment of sliding friction

Horizontal sled forces were measured using a load cell with a custom setup using methods previously described in the literature (25). Before testing, the load cell was calibrated, and the coefficient of sliding friction between the indoor synthetic surface and sled was determined. The equipment consisted of the load cell, amplifier (PT100LC, PT Limited, Auckland, New Zealand), power source (AC Adapter model: AIL4542 M9636, Dick Smith, Melbourne, Australia), and CompactDAQ chassis (US9162, National Instruments, Texas, Untied States) connected to a data acquisition system (NI-9215, National Instruments, Texas, Untied States). Calibrations in the current study were calculated using a seven-point regression to ensure accuracy across a range of loads.

### Data collection

#### Barbell box squat

**Figure 1:**
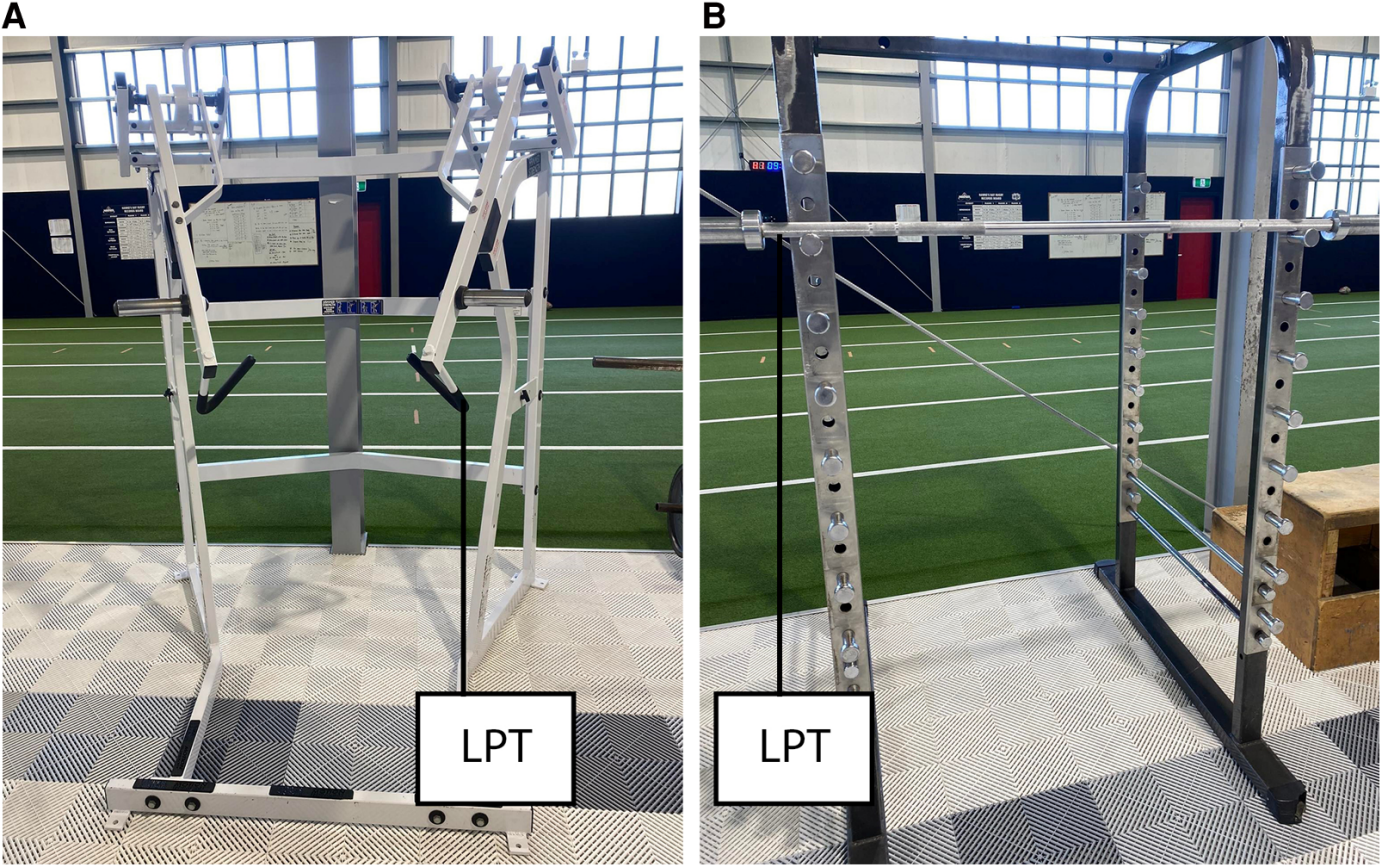
Testing set up for: (**A**) Double-arm jammer push-press and (**B**) Barbell box squat.

Before lifting, athletes were instructed to move the bar as fast as possible. To achieve maximal movement velocity, jumping, if possible, was permitted. Athletes’ squat depth was regulated by the use of a 52 cm box (see Figure 1). From a standing start, athletes were required to squat to touch the box and finish the repetition with maximal hip and knee extension for the repetition to be included. Self-selected bar position and foot placement were permitted. During each trial, velocity was recorded for each load for each athlete. To ensure a sufficient range of weights were lifted during testing, athletes lifted incrementally larger weights until they met a minimum velocity threshold of 0.7 m · s^−1^. A minimum of three loads were used to establish a force-velocity profile.

#### Double-arm jammer push-press

Athletes began the movement in a self-selected crouch position and were asked to propel the handles forward as fast as possible in one repetition (see Figure 1). Athletes were required to produce full elbow extension for the repetition to be included. The hip position was not specified to mimic the athlete's movement typically used in training and reduce any learning effect during testing. To ensure a sufficient range of weights were lifted during testing, athletes lifted incrementally larger weights until they met a minimum velocity threshold of 0.7 m · s^−1^. A minimum of three loads were used to establish a force-velocity profile.

#### Sled pull

**Figure 2:**
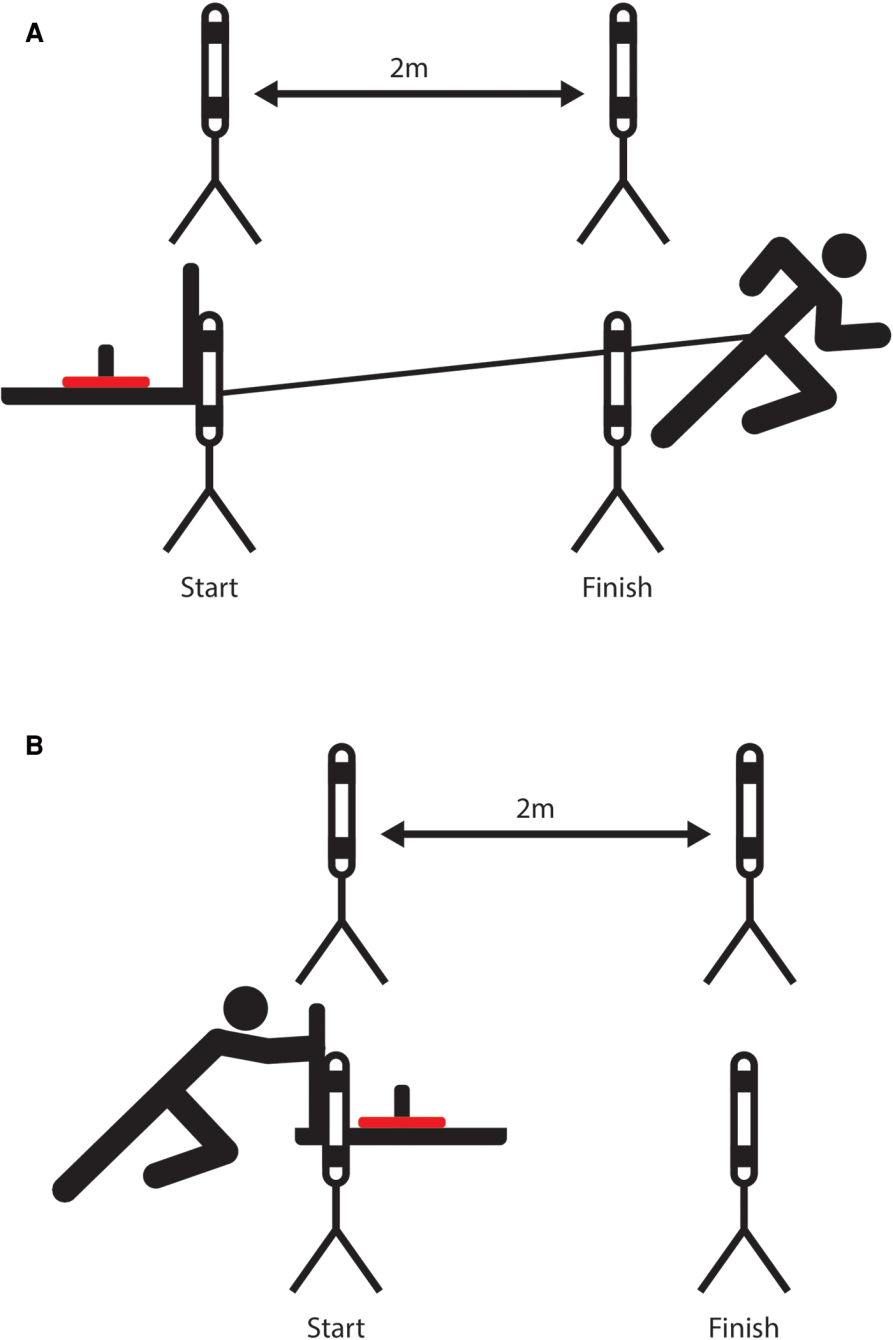
Testing set up for: (**A**) Sled pull and (**B**) Sled push.

Athletes were required to pull the sled over a 2 m distance. The sled (Xtreme Elite Prowler sled, Elite Fitness, Auckland, New Zealand) was positioned as close as possible to the first timing gate (Swift Duo, Swift Performance, Queensland, Australia) to ensure that the beam broke upon the initial movement of the sled. The athlete was connected to the sled *via* a harness and started from a position beyond the finish timing gate such that the time taken for the sled to move 2 m was recorded. A diagram of the sled pull setup is presented in Figure 2, part A. Athletes completed four incremental sled pulls with loads of 20 kg, 40 kg, 60 kg, and 80 kg, exclusive of sled weight (31 kg), consistent with methods previously described in a study conducted by Helland et al. (26).

#### Sled push

Athletes were required to push the sled over a 2 m distance in a setup similar to the sled pull (see Figure 2 part B). The sled was positioned to ensure the timing light beam would break at the initial push. Hand and foot positions were not standardised to reduce any learning effect. Athletes were reminded to push from the front foot and to ensure that the force was exerted horizontally. Four incremental sled push loads (exclusive of sled weight) were used; 20 kg, 40 kg, 60 kg and 80 kg.

#### Calculation of force-velocity profiles

**Figure 3:**
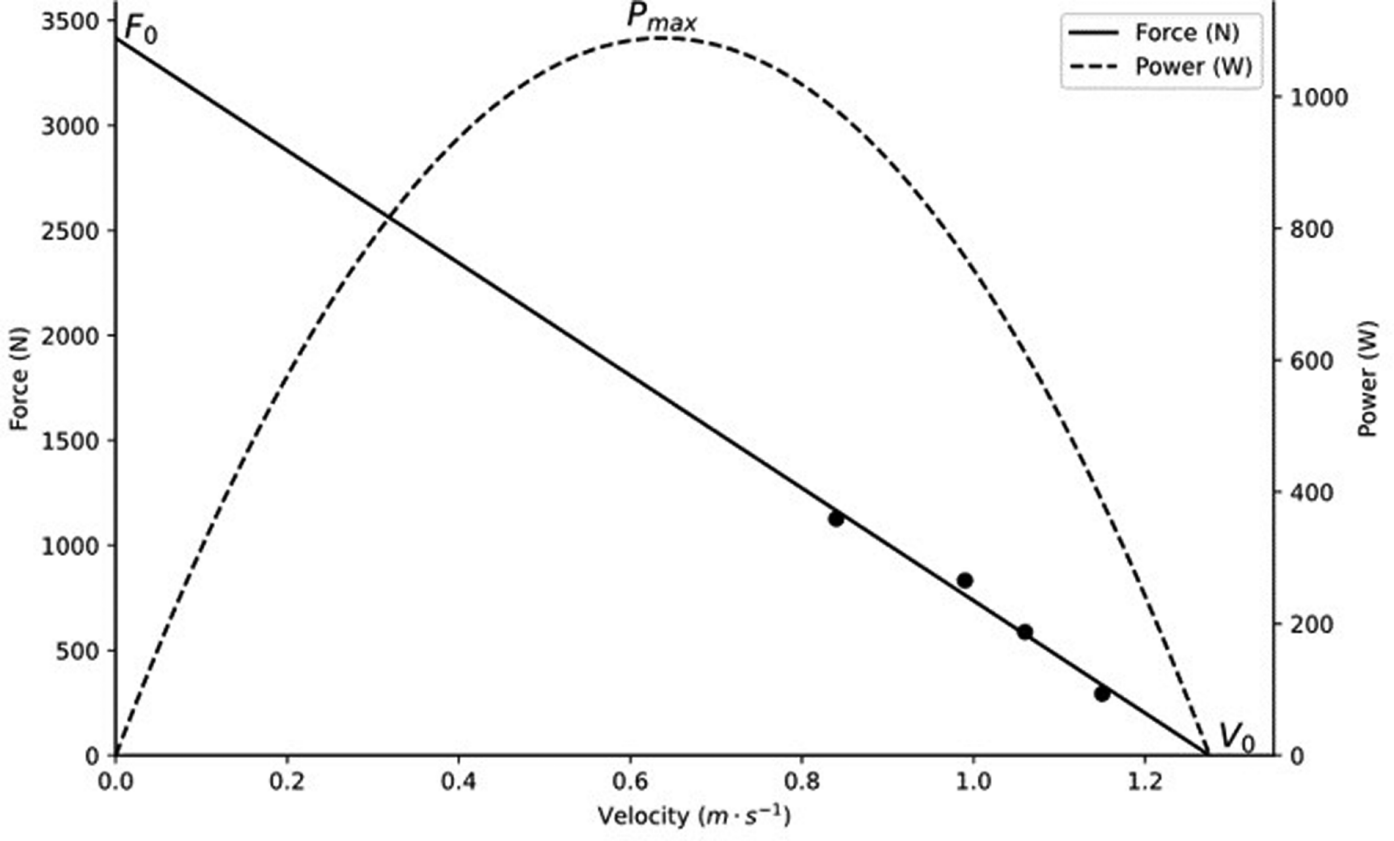
Example force-velocity profile.

FVPs were calculated in Microsoft Excel using formulas and mechanical variables described by Morin and Samozino (27, 28). See Figure 3 for an example box squat force-velocity profile.

#### Collection of match-day rugby performance indicators

Match-day statistics were collated from two trusted sources; the ESPN sports website and a Hawke's Bay Rugby Union performance analyst. Statistics on tries, turnovers, tackles, try assists, metres ran, defenders beaten and tackle breaks were acquired from the ESPN website as recorded by Opta Sports. Data from Opta Sports has been shown to demonstrate high reliability in football (29) and while previously used in Rugby Union analyses, to-date, there has been no published reliability of Opta Sports data within Rugby Union (30, 31). The Hawke's Bay Rugby Union performance analyst provided average post-contact metres only. Descriptions of each RPI are provided in Table 1. The entire Covid-impacted season of nine matches was included in the analysis, with FVP being conducted 12 days prior to the first match. To correct for variance in players game time over the season, each performance indicator was converted to values relative to game played. E.g., an athlete with 18 tackle-breaks in six matches scores identically to an athlete with three tackle-breaks in one match. The relative statistics for each player were used in statistical analyses.

**Table 1:** Performance indicators description.

Performance Indicator	Description
Post-contact metres	Metres are calculated from when the ball carrier makes contact with the opposition players until they are bought to the ground, go into touch, score a try, pass the ball or concede possession of the ball.
Tries	The number of tries a player has scored.
Turnovers conceded	The number of times the player has lost possession of the ball.
Tackles	The number of occurrences the players completed a successful tackle.
Try assists	The number of times a player was the assisting player that threw the final pass to the try scorer.
Metres ran	The total number of metres a player ran with the ball. This includes contact and non-contact carries.
Tackle-breaks	The total number of times an attacker had breached the defending line, including both contact and non-contact situations.
Defenders beaten	The number of occurrences evasive ball strategies (side-step or fend) caused a missed tackle for the defending team.

Performance measures are defined by Ungureanu (44) and Vanderpreet (personal communication, 2021).

### Statistical analyses

Analyses were performed using Python 3.10.4 and the packages pandas 1.4.1 and SciPy 1.8.0. A correlational analysis investigated the relationship between RPIs and FVPs of four common resistance exercises. A preliminary analysis was performed to check for assumptions of normality. Pearson's correlations were used where possible, whilst the Kendall Tau-B correlations were used for those that violated the assumption of normality. Alpha was set to *α* = 0.05. Correlations were described using Hopkins' qualitative descriptors (32).

Players that did not play any matches were removed from the analysis. In addition, players who did not have sufficient data to create FVPs were also excluded. These exclusions resulted in the eighteen players being included in this analysis.

Pearson's correlations were calculated between FVP variables across different exercises. The purpose of this was to determine the extent of relationship between exercises, with the aim of identifying any potential redundancies in measuring all exercises in future research. The findings showed no significant correlation between exercises, suggesting that the data obtained from each exercise is distinct. These results are presented as Supplementary Data.

## Results

### Force-velocity profile results

The athlete's FVP represents the mechanical properties of their neuromuscular system in the four common resistance tests. The mean FVP measures of the team in each of the four common resistance tests are presented in Table 2.

**Table 2:** Team force-velocity data.

Exercise	*F*_0_ (*N*)	*S_fv_*	*V*_0_ (m·s^−1^)	*P*_max_ (W)
Back squat	2,987.0 ± 563.6	−2,019.9 ± 503.6	1.5 ± 0.2	1,115.0 ± 198
Jammer push-press	1,762.0 ± 357.4	−810.3 ± 222.9	2.2 ± 0.3	978.2 ± 227.6
Sled push	597.8 ± 126.2	−212.4 ± 60.5	2.9 ± 0.4	427.6 ± 79.4
Sled pull	454.2 ± 76.7	−106 ± 97.6	3.7 ± 0.8	402.2 ± 30.1

Data is presented as Mean + SD. *F*_0_, theoretical force maximum; *S_fv_*, slope of the linear force-velocity relationship; *V*_0_, theoretical velocity maximum; *P*_max_, maximal power.

### Match-day rugby performance indicators

Summary data from the eight RPIs collected during match-day performances are collated in Table 3. Over the nine-match season, each player completed a mean of 6.5 ± 2.5 matches.

**Table 3:** Performance indicators data.

Match Statistic	Mean ± SD
PCM (m)	1.93 ± 0.99
Tries	0.2 ± 0.2
Turnovers	0.6 ± 0.6
Tackles	4.5 ± 2.5
Try assists	0.1 ± 0.2
Metres ran (m)	23 ± 19.1
Defenders beaten	1 ± 0.8
Tackle-breaks	1.5 ± 1.9

Data is presented as Mean + SD. PCM, post-contact metres.

### Correlational analyses

Table 4 shows correlations between measures from the FVPs and the RPIs. There was a statistically significant, moderate, positive correlation between tackle-breaks and sled push *V*_0_ (*r* = .35, *p* = .048). Large, positive correlations between tackles and jammer push-press *V*_0_ (*r* = .53, *p* = .049), and tackle-breaks and sled pull *F*_0_ (*r* = .53, *p* = .03). A large, negative relationship was found between sled pull *V*_0_ and tackle-breaks (*r* = −.49, *p* = .04). The largest correlation reported was seen between metres ran, and sled pull *F*_0_ (*r* = .66, *p* = .03).

**Table 4:** Correlations between rugby performance indicators and neuromuscular performance of four exercises.

RPI	PCM (m)		Tries		Turnover		Tackles		Try assists		Metres ran (m)		Defenders beaten		Tackle-breaks	
	Correlation	*p*	Correlation	*p*	Correlation	*p*	Correlation	*p*	Correlation	*p*	Correlation	*p*	Correlation	*p*	Correlation	*p*
BS_*F*_0_	0.00	0.99	0.04^†^	0.84	−0.17^†^	0.34	−0.14	0.59	0.06^†^	0.76	−0.16	0.52	−0.05	0.84	−0.24^†^	0.17
BS_S*_fv_*	−0.09	0.71	−0.04^†^	0.84	−0.07^†^	0.67	−0.22	0.38	0.19^†^	0.32	−0.20	0.43	−0.08	0.75	−0.20^†^	0.25
BS_*V*_0_	0.19	0.45	0.24^†^	0.19	0.01^†^	0.97	0.25	0.33	−0.27^†^	0.15	0.13	0.60	0.07	0.78	0.25^†^	0.15
BS_*P*_max_	0.12	0.63	0.06^†^	0.72	−0.22^†^	0.21	0.01	0.96	−0.22^†^	0.24	−0.07	0.77	0.00	0.99	−0.05^†^	0.76
JP_*F*_0_	−0.25	0.39	−0.34	0.24	−0.33^†^	0.11	0.14	0.62	−0.39^†^	0.07	−0.35	0.22	−0.34	0.24	−0.11^†^	0.58
JP_*S_fv_*	−0.25	0.38	−0.24	0.41	−0.30^†^	0.14	−0.29	0.32	−0.42^†^	0.052	−0.29	0.32	−0.23	0.44	−0.07^†^	0.74
JP_*V*_0_	0.02	0.94	−0.11	0.71	0.01^†^	0.96	0.53	0.049[Table-fn table-fn6]	0.04^†^	0.86	0.00	0.99	−0.11	0.70	−0.09^†^	0.66
JP_*P*_max_	−0.10^†^	0.67	−0.24^†^	0.24	−0.35^†^	0.09	0.19^†^	0.39	−0.32^†^	0.14	−0.25^†^	0.23	−0.26^†^	0.21	−0.13^†^	0.51
Sps_*F*_0_	0.02	0.93	0.15^†^	0.41	−0.11^†^	0.52	−0.16	0.52	−0.12^†^	0.52	−0.03	0.91	0.10	0.70	−0.21^†^	0.22
Sps_*S_fv_*	0.07	0.77	−0.04^†^	0.84	0.21^†^	0.24	0.27	0.28	0.14^†^	0.46	0.15	0.55	0.02	0.94	0.29^†^	0.09
Sps_*V*_0_	0.05^†^	0.76	−0.04^†^	0.84	0.16^†^	0.38	0.33^†^	0.06	0.06^†^	0.76	0.22^†^	0.23	0.14^†^	0.42	0.35^†^	0.048
Sps_*P*_max_	0.12	0.64	0.18^†^	0.33	−0.05^†^	0.79	0.02	0.93	−0.19^†^	0.32	0.14	0.57	0.23	0.37	0.03^†^	0.88
Spll_*F*_0_	0.10	0.77	0.12	0.72	0.43^†^	0.07	−0.42	0.19	0.37^†^	0.15	0.66	0.03[Table-fn table-fn6]	0.55	0.08	0.53^†^	0.03[Table-fn table-fn6]
Spll_*S_fv_*	0.05^†^	0.88	−0.08^†^	0.75	−0.13^†^	0.58	0.45^†^	0.06	−0.09^†^	0.72	−0.42^†^	0.09	−0.28^†^	0.24	−0.20^†^	0.45
Spll_*V*_0_	−0.16	0.65	−0.12	0.73	−0.28^†^	0.24	0.58	0.06	−0.42^†^	0.10	−0.58	0.06	−0.53	0.09	−0.49^†^	0.04[Table-fn table-fn6]
Spll_*P*_max_	−0.05	0.88	0.18	0.60	0.13^†^	0.58	0.51	0.11	−0.14^†^	0.58	−0.09	0.80	−0.18	0.59	−0.16^†^	0.54

All correlations are reported as coefficients unless stated otherwise. BS, barbell box squat; JP, jammer push-press; Spll, sled pull; Sps, sled push; PCM, post-contact metres; *F*_0_, theoretical force maximum; *S_fv_*, slope of the linear force-velocity relationship; *V*_0_, theoretical velocity maximum; *P*_max_, maximal power; RPI, rugby performance indicator.

^†^ Kendall Tau-B (*τ_b_*).

* *p* < .05.

## Discussion

### Force orientation

This exploratory study investigated the relationship between athlete neuromuscular performance derived from force-velocity profiles (FVPs) of four common resistance exercises and rugby performance indicators (RPIs). It is acknowledged that several factors contribute to RPI's, including but not limited to technical proficiency (5) and aerobic and anaerobic fitness (33). However, this study explicitly explores the relationship between FVPs and RPIs. Whilst large significant correlations were identified between some variables, notably, the study did not report any significant correlations between barbell box squat FVPs and any of the RPIs. Although vertical resistance training is common and beneficial for performance (9, 10, 34), the barbell box squat did not report any significant correlation in this study. The dissimilarities in force orientation with horizontal RPIs may explain the lack of relationship in the present study. Indeed, the literature has previously shown that elite rugby union players are required to produce force across a range of movement velocities to cope with the varied demands of rugby, which predominantly occur in the horizontal plane (1). However, horizontal propulsive force generation is a quality that traditional resistance training often fails to simulate (23). Thus, the findings of this study support those of Randell and colleagues (23), who also showed that horizontal resistance training demonstrated a significant and moderate relationship with RPIs, such as tackle-breaks, tackles, and metres ran, whereas vertical resistance training did not.

The largest correlation in the current study was identified between sled pull force and metres ran in a match (*r* = .66, *p* = .03), which both occur in the horizontal domain. These findings are indicative of a relationship between the two variables; the magnitude of this correlation may be attributed to the initial acceleration from a stationary position. Indeed, initial acceleration is something all players must be good at to perform at a semi-professional level, due to the minimal distance between attackers and defenders (35). In other sports that require sprinting, the importance of rapid horizontal force production is also apparent, where one of the differentiating points between elite and sub-elite sprinters is the elite's ability to produce higher horizontal forces at any given moment (36). The ability to generate higher propulsive forces seen in the present study may be related to metres run in a non-contact scenario as it would enable attackers to accelerate away from defenders, resulting in players being able to cover greater distances. In contrast, the ability of attackers to generate force in a contact scenario may assist players with the leg drive that subsequently enables them to break tackles (7, 8), and, consequently, travel more metres. Since the findings of this study should be considered exploratory, further research is needed to determine the benefits of establishing sled pull FVPs on metres run during a match performance and the relevance of this conditioning in contact and non-contact scenarios.

### Sled pull and tackle-breaks

The present study found that the sled pull *F*_0_ (*r* = .53, *p* = .03) and sled pull *V*_0_(*r* = −.49, *p* = .04) demonstrated significant, large correlations with the number of tackle-breaks in the season, such that athletes with greater force dominant sled pull FVPs recorded a greater number of tackle breaks. The large negative correlation between sled pull velocity and tackle breaks further supports this relationship. This negative correlation suggests that the less velocity-focused the athlete, the greater the number of tackle breaks.

Considering neuromuscular abilities alone, the defining factor in post-tackle success is the ability of one player to exert greater force than the opposition. In a game, this could refer to the attacker actively trying to break free of the tackle by “pulling” away from the defender (termed the “pull contact”).

**Figure 4:**
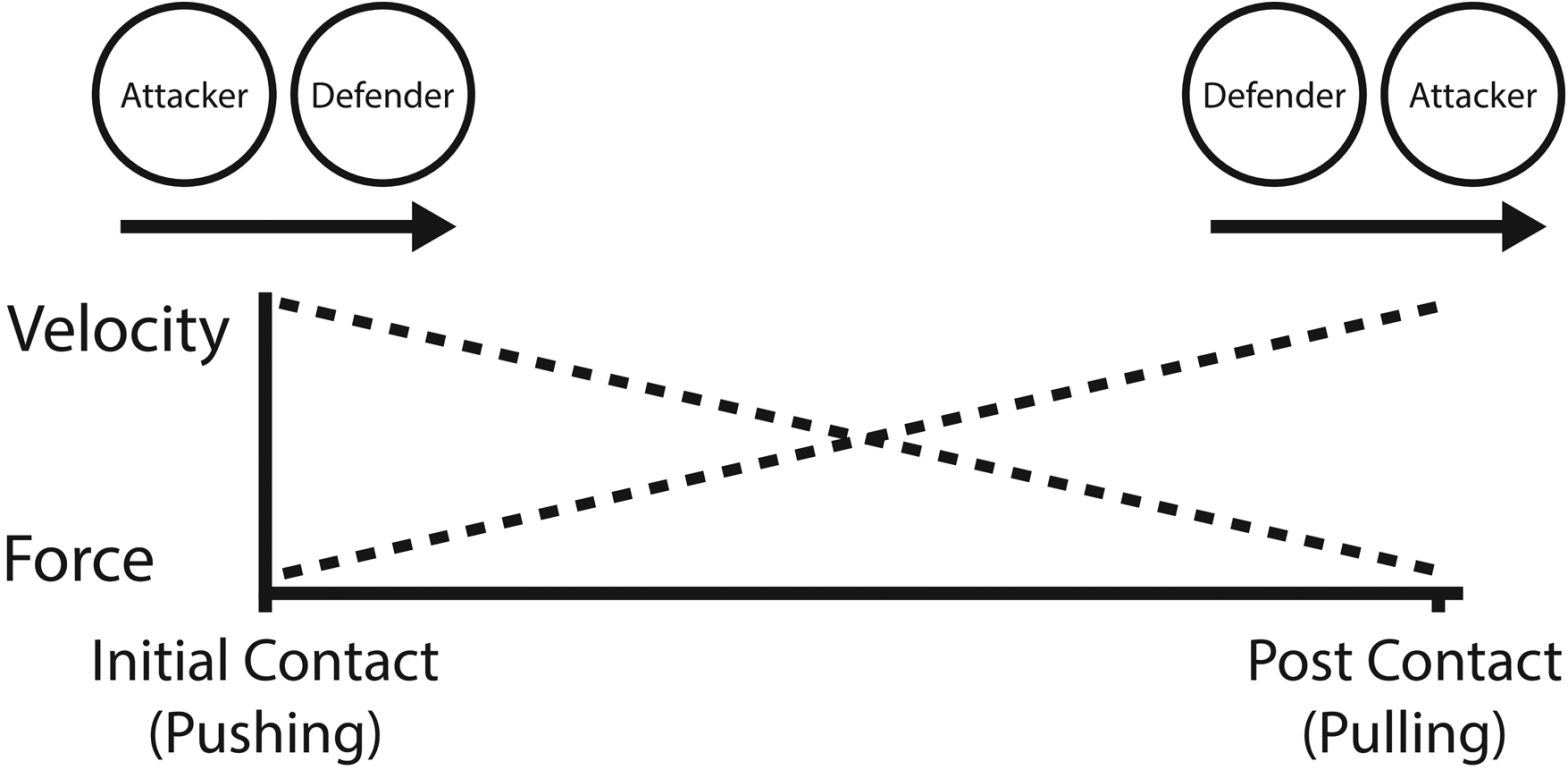
Hypothetical force and velocity demands during tackle contests.

The results from this study suggest that high sled force and low sled velocity may be related to tackle-breaks at different instances throughout the tackle contest (see Figure 4), which aligns with findings from other sources related to dominating the tackle contest (5, 7, 11, 12). This may be due to similarities between the pull movement in the sled pull conditioning activity and the pull-contact phase associated with breaking tackles in match-day performances. These preliminary findings are presented with caution due to the exploratory nature of this study. Subsequent studies should further investigate these findings.

In contrast to the importance of low-velocity FVPs in sled pull, the findings of this study suggest that players who display more velocity-dominant sled push tend to make more tackle-breaks (see Figure 4). The present study found that the sled push *V*_0_ demonstrated a moderate relationship with tackle-breaks (*r* = .35, *p* = .048). The greater correlation between the sled pull *F*_0_ and tackle-breaks than between sled push *V*_0_ may be related to the fact that the arms can move freely during the sled pull, which may better replicate sprinting mechanics (37). In contrast, in sled push conditioning, the arms are locked to exert the pushing force. In match day performance, for ball carriers to be successful, they must demonstrate “explosiveness on contact”, provided they are technically competent (5). The velocity of the tackle contest enhances momentum, which is necessary to dominate the contest. Indeed, the minimal distance between players may explain why velocity is vital (35). These findings agree with Lockie and colleagues (38), who suggest that velocity is integral for team sports like rugby. Specifically, the initial contact that could potentially create a tackle-break. Wheeler and Sayers (7) also support the present findings, whereby players in their study who received the ball at higher velocities dominated the tackle contest. The findings of this study indicate that sled conditioning may enhance physical attributes related to tackle-breaking ability. Nonetheless, it is important to note that these results are preliminary, and more extensive, long-term studies are required to fully verify the hypothesis that sled conditioning leads to an improvement in tackle-breaking performance.

The similarities of horizontal force orientation and low body positions seen in both sled pull and push may explain the greater correlation between these conditioning exercises than the other exercises employed in the study. For an attacker, the resistive force, i.e., defender, is effective in dragging the player backward during the tackle contest. Thus, the player must exert high horizontal forces to go past the defender and create a tackle-break (7). Conversely, in the sled push, the load is positioned anterior to the athlete and requires a horizontal force production to move the sled (40, 41). The restriction of arm movement in the conditioning exercise used in this study may have limited the ability of the player to exert optimal force thus, the relationship was more significant in the velocity end of the FVP. Irrespectively, the similarities of low body positions and force orientation in conditioning activities must be acknowledged to apply the importance of the conditioning principle of specificity.

### Conditioning for making tackles

Beyond the sled-based exercises, the only other significant and large correlation observed in this study was between jammer push-press *V*_0_ (*r* = .53, *p* = .049) and tackles. Of the four chosen exercises, the jammer was the only one that incorporated the upper and lower limbs. Despite the well-established importance of a strong leg drive for tackling success (7, 11, 31, 35), this was the only conditioning movement to demonstrate a relationship with tackles made. The finding from this study is supported by those from Speranza and colleagues (9), who showed that tackling ability correlated with upper limb movements (3RM bench press *r* = .72) and speed-strength (plyometric push-up, *r* = .70) in semi-professional rugby league players. Tierney and colleagues (5) reported a similar finding in rugby union, where upper-body movements significantly influenced tackling ability.

The present study results agree with others in suggesting that pushing velocity may enhance a defenders ability to execute a successful tackle (5, 7, 8, 11, 12). More specifically, we showed that velocity-dominant jammer push-press profiles correlate well with the number of tackles made, and sled push velocity correlates well with tackle breaks made. However, further longitudinal and experimental research is needed to confirm this. Velocity into the tackle increases a player's momentum, and as we, and others, have previously identified, momentum is vital to dominate the tackle contest (11). Hendricks, Karpul, et al. (11) reported that defenders dominated 57% of the tackle contest despite being at a mass disadvantage. Their success was attributed to entering the tackle contest at higher velocities than the attacker. Tierney et al. (5) reported similar results, where explosiveness on contact was related to the success of the tacklers. By contrast, it should be noted that the same research group reported conflicting results, where velocity was not a determinant of tackling success (41). For the reasons already identified in this study, it is easy to see how assuming performance outcomes based purely on neuromuscular mechanical abilities alone may result in contrasting conclusions. To the best of our knowledge, this exploratory study is the first to describe a relationship between the jammer push-press and a rugby performance indicator; we, therefore, encourage further research into this conditioning movement.

### Maximal power vs. speed-strength for performance indicators

Previous research has emphasised the importance of speed-strength to break tackles during the contact phase of the tackle contest or for evasive strategies such as side-stepping (42, 43), using phrases such as “explosiveness on contact” (5). One component of speed-strength is *P*_max_ however, the results of the present study found no significant correlation between *P*_max_ and any of the RPIs identified. Nevertheless, we acknowledge that the definition of *P*_max_ explicitly refers to the mechanical definition of power (force × velocity), whereas speed-strength refers to the entire force-velocity spectrum, including the point of *P*_max_. Therefore, these results suggest training for *P*_max_ may be less optimal than training at a specified balance of force and velocity for a given exercise and RPI. Further research should investigate these notions to confirm the exploratory results found in this study.

### Limitations

As with all research, this study has limitations that must be considered while interpreting the results. Firstly, the correlational design of the study, with a multitude of comparisons, increases the risk of Type I error. Consequently, it is imperative to acknowledge that this study is exploratory, and its primary objective is to provide insights that can inform future studies to test specific hypotheses derived from the findings.

Secondly, the study found correlations between certain FVP variables and RPIs, suggesting that targeting training to improve FVP variables may be effective for enhancing Rugby performance. However, it is essential to note that this study was not designed as an intervention study, and further research should be conducted through interventional designs to determine the training implications of these results and assess the chronic adaptations.

In addition, the sample size was smaller than anticipated due to availability constraints among the players. The original sample consisted of 22 athletes but was initially reduced due to player injuries. Several high-profile players were also unavailable due to external professional commitments, further limiting the sample size.

Finally, it is worth noting that this study represents the first attempt to create FVPs in the jammer push-press. The design of the jammer equipment presents a challenge in accurately measuring velocity due to its restricted movement along an angled arc. The LPT used in this study employs angle correction to adjust for variations in lifting position, thereby restricting measurements to the vertical plane. It was hypothesised that LPT measurements along the vertical plane would be proportional to measurements taken along the entire arc of the jammer exercise. However, further research is necessary to fully understand the effects of LPT placement on jammer exercise measurements.

### Practical applications

**Figure 5:**
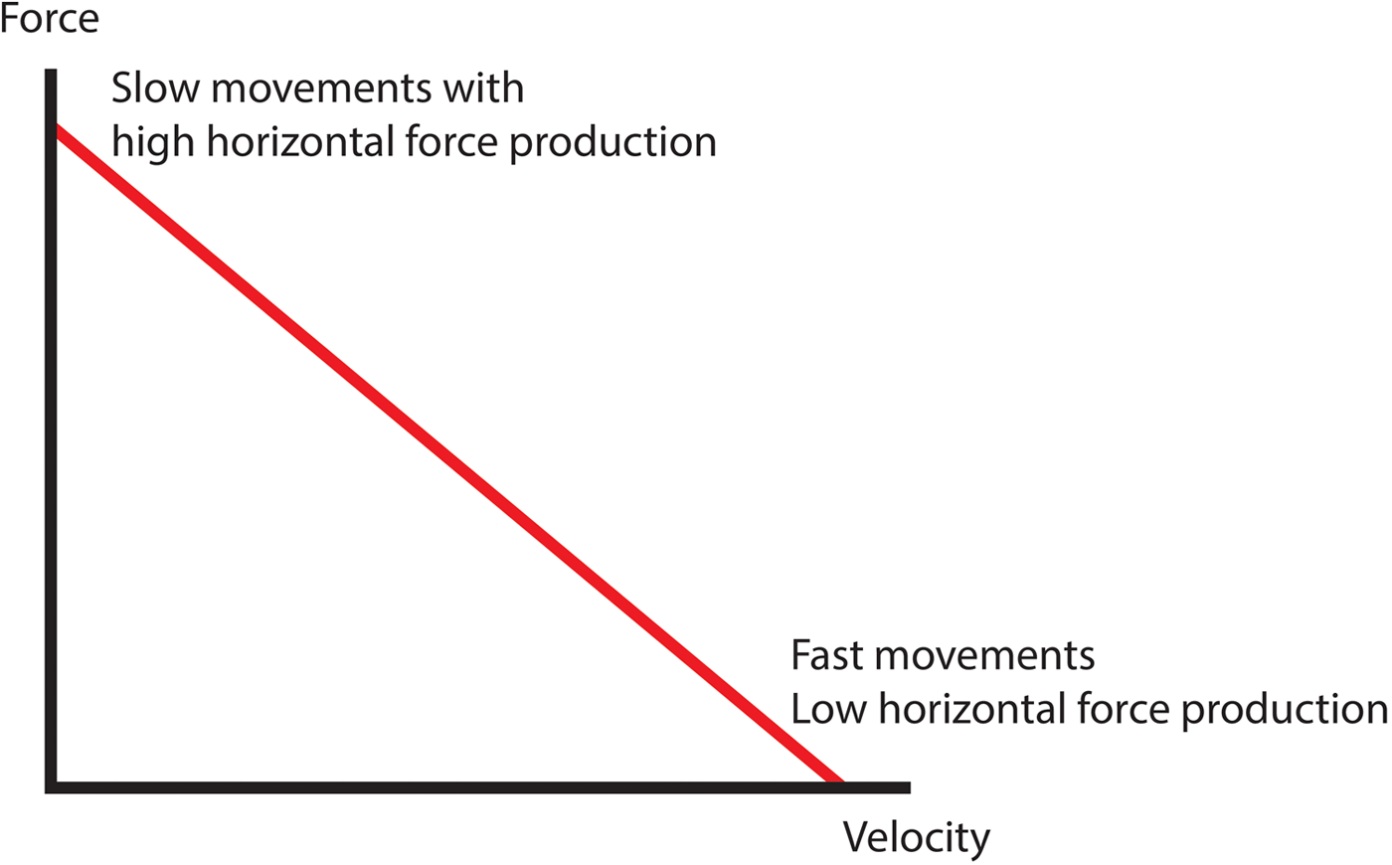
Proposed relationships between on-field demands and force-velocity profiles.

To provide a graphical summary of these exploratory findings, Figure 5 shows how FVPs could be used to enhance rugby performance indicators. Our results suggest that pushing sled loads at high velocities relates to the number of tackle-breaks a player makes. Ultimately, our results imply light sled loads should be used to promote high-velocity pushing movements should be prescribed to enhance tackle-breaks.

The present study suggests that pulling force is important in contact and non-contact scenarios. Therefore, it is recommended that coaches employ high-load sled-pulling exercises to prepare athletes to break free from a tackle and create space between themselves and defenders during the initial acceleration period.

Coaches that wish to create more tackle-breaks at contact may consider light-load sled pushing. The load should be light enough to encourage high-velocity movements. Doing so likely maximises horizontal force production to reach maximal speeds.

Finally, the jammer push-press demonstrated the largest relationship with tackles made. This suggests a combination of lower and upper limb pushing movements performed at high velocities may be useful for training tackling. The similarities between the jammer push-press and tackling concerning force orientation and movement velocity may explain the large relationship. Coaches that want to increase the number of tackles made may benefit from incorporating movements like the jammer push-press at high velocities.

## Conclusion

This exploratory study highlights the importance of exercise specificity, such as exercise orientation during prescription. Although traditional vertical resistance training is valuable, the transference to the field may be hindered due to a lack of specificity. Therefore, coaches should consider incorporating horizontal resistance training into rugby programmes. Furthermore, FVP is a valuable assessment tool that provides detailed insight into an athlete's force and velocity capabilities. Profiles should be examined in conjunction with RPIs and positional demands to inform resistance training prescription better.

## Data Availability

Further analysis is provided in the supplementary data, in addition, the raw data supporting the conclusions of this article will be made available by the authors on request, without undue reservation.
